# 1553. Cost Effectiveness of Cabotegravir versus Continuous and Event-Driven PrEP: A Systematic Review and Cost-Analysis

**DOI:** 10.1093/ofid/ofad500.1388

**Published:** 2023-11-27

**Authors:** Ishani Sharma, Andrew Hill

**Affiliations:** Imperial College London; University of Liverpool, London, England, United Kingdom

## Abstract

**Background:**

The HIV epidemic continues to grow with 1.5 million new infections globally in 2021. In 2019, 62% of new infections were amongst key at-risk populations. HPTN 083 and 084 trials showed up to 88% increased efficacy of long-acting cabotegravir (CAB-LA) compared to continuous oral tenofovir/emtricitabine (TDF/FTC). However, the expense of CAB-LA ($22,200 per person per year (pppy)) limits its availability to populations who need it most. In most countries, HIV prevention budgets are highly limited, with TDF/FTC available as a low-cost generic.

**Methods:**

We conducted a systematic review of studies on global HIV incidence in at-risk populations. The weighted incidence was calculated for each population, blood donors, and the general population. We evaluated potential HIV infection rates for 4 prevention strategies: no PrEP, continuous CAB-LA (annual cost $22,200 per person (pp)), continuous TDF/FTC (annual cost $48pp), and event-driven TDF/FTC (annual cost $12pp). For each PrEP strategy we assumed an additional $4 pppy for HIV testing, and $14 pppy for education and service access. Assumed efficacy was 90% for continuous CAB-LA, 60% for continuous TDF/FTC, and 30% for event-driven TDF/FTC. Using weighted HIV incidence rates and an assumed $1million fixed budget for HIV prevention, annual HIV infection rates for each target population were calculated for each PrEP strategy.

**Results:**

The database searches identified 98 studies in 5,230,189 individuals. Incidence per 100 person-years (pys) ranged from 0.03 in blood donors to 3.82 in people who inject drugs (Table 1). Within the fixed $1 million budget, annual HIV infections in commercial sex workers (incidence of 3.1/100pys) would be 1,033 for no PrEP, 1,032 for continuous CAB-LA, 752 for continuous TDF/FTC, and 723 for event-driven TDF/FTC. The same trends were seen across all populations: use of event-driven PrEP prevented the largest number of HIV infections for fixed budgets.

Table 1

**Figure:**
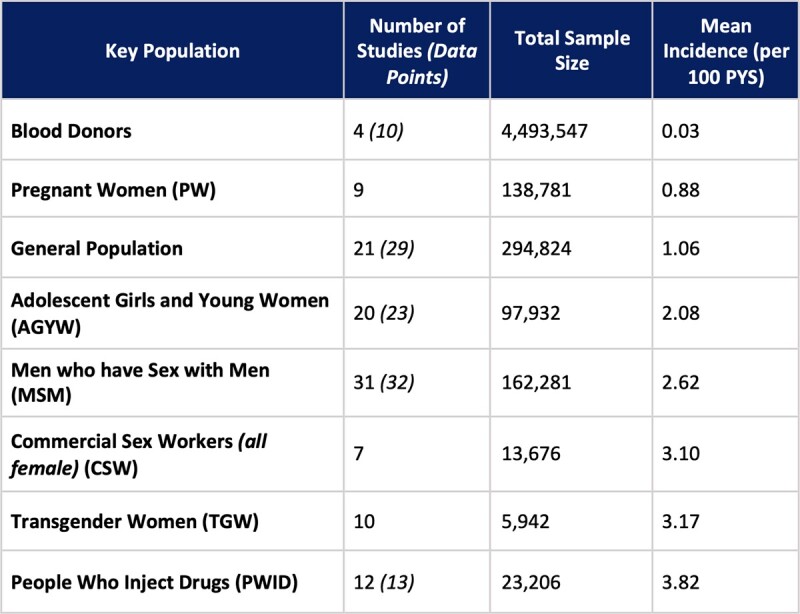

A table showing the number of studies, total sample size, and average incidence of HIV infection in key populations, blood donors and the general population.

**Conclusion:**

CAB-LA is the most efficacious form of PrEP, but high prices limit numbers who can be treated. More HIV infections can be prevented using low-cost event-driven TDF/FTC as PrEP: far more people can be treated for fixed budgets. This result is consistent across a range of at-risk populations in different countries.

**Disclosures:**

**All Authors**: No reported disclosures

